# MDEFusion: A Multi‐Domain EEG Feature Fusion Network With Bidirectional Attention LSTM and Half‐Split Crossover SAE for Schizophrenia Recognition

**DOI:** 10.1002/brb3.71315

**Published:** 2026-04-22

**Authors:** Xiaofeng Li, Heyan Huang

**Affiliations:** ^1^ School of Computer Science and Technology Beijing Institute of Technology Beijing China; ^2^ Southeast Academy of Information Technology Beijing Institute of Technology Putian Fujian China

**Keywords:** bidirectional long short‐term memory, electroencephalogram, multi‐domain feature fusion, schizophrenia recognition, sparse autoencoder

## Abstract

**Purpose:**

Schizophrenia (SZ) is a severe mental disorder. Using electroencephalogram (EEG) signals for objective and accurate recognition of SZ is critical for timely clinical intervention. However, due to the highly non‐stationary nature of EEG signals and the complex spatial correlations of neural activities, existing recognition methods still face key challenges, including incomplete feature extraction, limited computational efficiency, and insufficient modeling of long‐range temporal dependencies.

**Methods:**

To address these issues, this paper proposes a multi‐domain EEG feature fusion network (MDEFusion). By synergistically introducing a half‐split crossover sparse autoencoder (HCSAE) and a bidirectional attention long short‐term memory (BALSTM) network, the model achieves efficient fusion of multi‐domain features and the modeling of long‐range temporal dependencies. Specifically, MDEFusion constructs an improved SAE with a half‐split crossover mechanism to perform nonlinear cross‐fusion of features across the time, frequency, and spatial domains. This effectively compresses high‐dimensional redundant information while simultaneously enhancing cross‐domain feature interaction capabilities. Furthermore, the BALSTM network is utilized to strengthen the contextual correlation between forward and backward sequences. This enables the precise capture of subtle yet critical pathological dynamic features in EEG signals, effectively mitigating the difficulty of stably modeling long‐period temporal dependencies.

**Results:**

Experimental results demonstrate that, compared with state‐of‐the‐art methods, MDEFusion achieves an accuracy of 93.1% on the RepOD dataset and 94.6% on the NNCI dataset.

**Conclusion:**

This paper provides an efficient and reliable EEG analysis tool for the auxiliary diagnosis of schizophrenia, demonstrating significant application value for clinical decision support systems.

## Graphical abstract

This paper proposes a multi‐domain EEG feature fusion network. By synergistically introducing a half‐split crossover sparse autoencoder and a bidirectional attention long short‐term memory network, the model achieves efficient fusion of multi‐domain features and modeling of long‐range temporal dependencies.

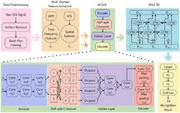

## 1 Introduction

In the fields of cognitive science and neurophysiology, human brain activity has been proven to play a vital role in physiological and emotional processes. Electroencephalogram (EEG) signals, as the core means of capturing these dynamic changes, have become a prerequisite for the monitoring and objective diagnosis of mental disorders such as schizophrenia (SZ) (Uhlhaas and Singer 2010; Lawhern et al. 2018). However, clinical recognition tasks for SZ face significant inherent challenges (Sarisik et al. 2025; Gordillo et al. 2023; Chen et al. 2024). These primarily stem from the multi‐dimensional heterogeneous attributes of EEG signals (Latreche et al. 2024), where pathological features are intertwined across the temporal, frequency, and spatial domains (Graña and Morais‐Quílez 2023). Furthermore, the large volume of data generated by high sampling rates imposes strict requirements on processing efficiency, and the strong non‐stationarity of the signals makes long‐period dynamic temporal dependencies extremely difficult to capture stably (Nikulin et al. 2012; Saha and Baumert 2020). To address these challenges, existing methods can be classified into three categories: (1) Signal decomposition‐based analysis methods: These primarily focus on feature extraction in the time‐frequency domain but often overlook spatial correlations, leading to incomplete modal representation (Aziz et al. 2024). (2) Feature engineering and deep feature representation architectures: While these can improve performance on small‐scale samples, their feature analysis techniques are relatively singular and struggle to cover the global information of EEG signals (Shoeibi et al. 2021; Zhang et al. 2020). (3) Attention‐based spatiotemporal modeling methods: Although these strengthen feature correlations, the model structures are often overly complex, resulting in a distinct weakness regarding the computational efficiency required for clinical deployment (Bagchi and Bathula 2022). The deep heterogeneity of multi‐domain features in SZ recognition tasks, combined with the urgent clinical need for low overhead, means that current methods struggle to maintain a logical balance between global feature fusion, long‐range dependency mining, and computational light‐weighting (Rahul et al. 2024; Qiu et al. 2018). This has left a gap in the availability of efficient auxiliary diagnostic tools that meet both performance and efficiency requirements. Consequently, this paper proposes MDEFusion. Its core logic utilizes a half‐split crossover sparse autoencoder (HCSAE) as a feature engine to perform deep nonlinear crossover and redundancy compression on the initially extracted time, frequency, and spatial features. Simultaneously, it integrates a bidirectional attention long short‐term memory (BALSTM) network to capture past and future temporal context information, aiming to achieve an efficient transformation from raw data to pathological discrimination through an end‐to‐end collaborative optimization architecture.

Our main contributions are as follows:

1. We propose an end‐to‐end MDEFusion that achieves collaborative optimization from multi‐domain EEG feature fusion to recognition. This significantly reduces computational complexity while maintaining high performance.
2. We proposed an HCSAE structure for multi‐domain EEG feature fusion. By designing a half‐split crossover module to facilitate feature fusion and jointly optimizing the activation functions and sparsification strategies, we improved the efficiency and accuracy of feature fusion.
3. We designed a BALSTM network to strengthen bidirectional temporal dependencies. Through the fusion of forward and backward sequence information guided by internal self‐attention, we enhanced the model's capability to represent long‐term dependency patterns in EEG signals.

## 2 Related Works

To address the challenge where signal decomposition analysis methods focus heavily on time‐frequency extraction while neglecting spatial domain correlations, leading to incomplete modal representation, researchers have primarily explored diversifying feature extraction (Shen et al. 2023). Khare et al. 2023 introduced the Margenau–Hill time‐frequency distribution to capture instantaneous spatiotemporal information; although this enhanced detailed representation, the conversion to 2D images significantly increased front‐end processing overhead. Building on this, and to reduce reliance on handcrafted features, Li et al., 2023 proposed mapping 1D signals into 3D images and utilizing vision transformers to fully preserve the spatiotemporal characteristics of EEG; however, the demand for computational resources increased exponentially with the increase in dimensionality. These studies reflect an evolution from single‐domain analysis toward multi‐dimensional mapping, but the problem of efficiently mining spatial correlations remains unresolved. To this end, the MDEFusion framework proposed in this paper integrates wavelet packet transform (WPT) and independent component analysis (ICA), aiming to achieve deep mining and preliminary mixing of features across temporal, frequency, and spatial dimensions simultaneously, effectively overcoming the bottleneck of incomplete modal representation. To address the singular nature of feature analysis techniques in architectures based on feature engineering and deep feature representation, as well as the insufficient coverage of deep latent features, related research has focused on improving the discriminative power of models (Li et al. 2023; Kumar et al. 2023). Ruiz de Miras et al. 2023 utilized various nonlinear metrics combined with PCA dimensionality reduction to enhance model stability, yet remained limited by the efficiency of manual feature selection. To further automate feature refinement, Jing et al. 2024 proposed an effective information estimation framework, which enhances feature discriminability through a feedback mechanism between 1D and 3D convolutional neural networks. While these methods have improved feature selection efficiency, bottlenecks remain, such as insufficient enhancement of the temporal dimension. To address these limitations, this paper proposes a HCSAE, which implements cross‐exchange between features via a half‐split crossover module. This reduces coupling between features while preserving internal information, achieving the organic fusion of multi‐domain features through convolutional refinement. To address the trade‐off between overly complex model structures and computational efficiency in attention‐based spatiotemporal modeling, research has pivoted toward topological optimization and correlation enhancement (Hussain et al. 2024). Zhu et al. 2022 proposed a divergence metric method that combines multiple information sources to increase analytical depth by modeling evidence differences; however, its massive computational load limits its practical utility. To balance efficiency, Hassan et al. 2023 attempted to reduce bandwidth consumption through three‐channel filtering, while Usman et al. 2024 further introduced dictionary learning and sparse coefficient solvers to achieve more precise channel feature selection, though this carries the risk of ignoring individual topological differences. These works highlight the necessity of balancing model complexity with performance. The proposed BALSTM in this paper utilizes internal self‐attention to guide bidirectional temporal modeling, enhancing the capture of long‐range pathological features. When integrated into the end‐to‐end MDEFusion framework, it significantly reduces computational complexity while maintaining high performance, achieving a deep alignment between high‐precision diagnosis and lightweight inference.

## 3 Methodology

### 3.1 Overall Framework

**FIGURE 1:**
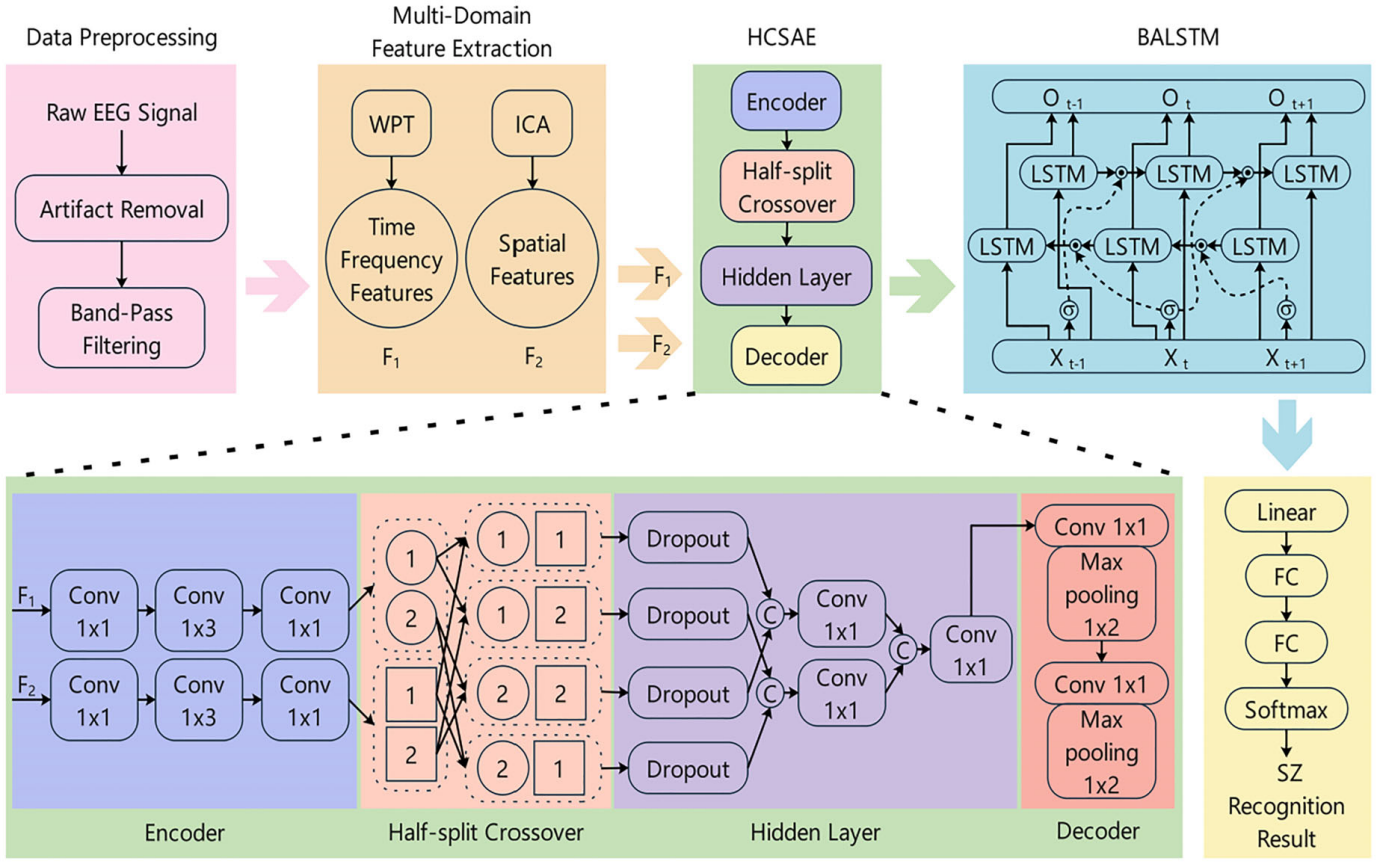
Framework for MDEFusion.

The processing of EEG signals typically leverages deep learning to analyze captured information for pattern recognition tasks, after which the recognition results are output to external devices to provide data support for clinical auxiliary diagnosis. EEG signals possess distinct domains; consequently, this paper first analyzes features across the time, frequency, and spatial domains. Subsequently, fusion and recognition are performed on the multi‐domain EEG signals. In the fusion and recognition analysis, improvements are implemented for both SAE and long short‐term memory (LSTM) networks. SAE performs excellently in the deep fusion of multi‐domain feature information, while LSTM networks can effectively learn the dynamic temporal information of EEG signals. The combination of these two neural networks facilitates the enhanced processing of multi‐domain EEG signals. The framework for MDEFusion is illustrated in Figure 1.

### 3.2 EEG Signal Preprocessing

#### 3.2.1 Datasets

The experimental data were obtained from two public datasets: the RepOD dataset (Olejarczyk and Jernajczyk 2017) and the NNCI dataset (Borisov et al. 2005). **RepOD dataset**: Participants in the dataset included 14 patients with paranoid SZ (7 males, average age 27.9 ± 3.3 years; 7 females, average age 28.3 ± 4.1 years) and 14 healthy controls (HC) (7 males and 7 females, corresponding average ages 26.8 ± 2.9 years and 28.7 ± 3.4 years). All subjects were required to remain in an eyes‐closed resting state during data collection. EEG signals were recorded using a standard 10–20 system equipped with 19 channels. **The Laboratory for Neurophysiology and Neuro‐Computer Interfaces (NNCI) dataset**: Regarding the dataset, all participants were boys, including 45 patients with SZ (aged 10 years 8 months to 14 years) and 39 HC (aged 11 years to 13 years 9 months). The average age for both groups was 12 years and 3 months. All clinical diagnoses were confirmed by experts from the Mental Health Research Center. For this dataset, EEG data were captured using a 16‐channel device adhering to the standard 10–20 configuration system.

To address the channel number mismatch between the RepOD (19 channels) and NNCI (16 channels) datasets, we adopted a channel mapping and interpolation strategy based on the standard 10–20 system. First, we identified the 16 standard electrode positions shared by both datasets (Fp1, Fp2, F3, F4, C3, C4, P3, P4, O1, O2, F7, F8, T7, T8, P7, P8). For the three additional channels in the RepOD dataset (FCz, CPz, Oz), we retained their data. For the NNCI dataset, we utilized spherical spline interpolation based on the 3D coordinates of the electrode sites to estimate the electrical signals at the FCz, CPz, and Oz positions from the original 16‐channel signals. Through this procedure, both datasets were unified into the same 19‐channel set, which was ultimately used for model training and testing. The specific channel list consists of Fp1, Fp2, F3, F4, C3, C4, P3, P4, O1, O2, F7, F8, T7, T8, P7, P8, FCz, CPz, and Oz.

#### 3.2.2 Preprocessing and Standardization Workflow

The acquisition of EEG signals is non‐invasive, and during the data acquisition process, it was first ensured that the equipment was properly grounded and situated in a quiet environment to avoid electromagnetic interference. A band‐pass filter was employed to limit the frequency range to 0–100Hz to preserve physiologically meaningful EEG rhythms. To address various types of noise and interference, such as artifacts, present in the EEG signals from the two public datasets, consistent data preprocessing was applied to ensure compatibility between the RepOD and NNCI datasets. Both datasets were normalized to a common scale to enable comparative analysis. Since the preprocessed signal lengths differed across datasets, EEG sequences were segmented into standardized temporal segments using a 2‐s window with a 1‐s stride. This windowing strategy guaranteed uniform input dimensions for subsequent feature extraction and model training. Statistics of the processed datasets are summarized in Table 1.

**TABLE 1:** Sample after EEG preprocessing.

Datasets	SZ group samples	HC group samples	Recording duration	Number of samples after segmentation	
				SZ group	HC group
RepOD	14	14	15 min	12,586	12,586
NNCI	39	39	1 min	2301	2301

#### 3.2.3 Dataset Partitioning

To address the class imbalance in the NNCI dataset, random under‐sampling was performed to retain 2301 representative samples from the SZ group. To prevent data leakage, a subject‐independent splitting strategy was employed: 80% of the subjects were assigned to the training set, while the remaining 20% were equally partitioned into validation and test sets (10% each). Within each subset, samples were shuffled to eliminate temporal dependencies. The detailed distribution of the partitioned data is summarized in Table 2.

**TABLE 2:** Dataset partitioning.

	Training set	Verification set	Test set	Total
1. RepOD	19,778	2697	2697	25,172
2. NNCI	3658	472	472	4602

After preprocessing the EEG signals, the time‐frequency and spatial‐domain features of the EEG signals are computed, and then input into the MDEFusion recognition model for computation and analysis. In MDEFusion, the BALSTM network has 64 nodes in the hidden layer. The decay rate is set to 0.9. The number of epochs is set to 200.

### 3.3 Multi‐Domain EEG Feature Extraction

In multi‐domain EEG signal recognition, the extraction of discriminative and informative features is a crucial step. This paper utilizes WPT and ICA methods to extract the time‐frequency and spatial domain features of EEG signals, which are highly suitable for analyzing non‐stationary biological signals. The wavelet transform extracts time and frequency domain information from EEG signals and captures time‐frequency transformation information. This method accurately partitions time at high signal frequencies and precisely partitions frequency at low signal frequencies, making it suitable for acquiring effective features from EEG signals. The EEG signal sequence is represented as follows:

(1) $$X\left(t\right)=\left\{\left.x_{1}\left(t\right),x_{2}\left(t\right),\text{…},x_{m}\left(t\right)\right\}\right.,i\in \left[1,m\right]$$

where $x_{i}\left(t\right)$ represents the EEG signal vector, t is the time point. m is the sample size.

The equation for extracting time‐frequency domain features of EEG signals using the wavelet transform is as follows:

(2) $$F_{1}=\frac{1}{\sqrt{\beta }}\int_{-\infty }^{\infty }X\left(t\right)W\cdot \left(\frac{t-\chi }{\beta }\right)dt$$

where β represents the scale factor, χ represents displacement, W(·) represents a wavelet function. $F_{1}$ is the time‐frequency domain features of the EEG signal obtained after the wavelet transform.

The Equation for extracting spatial domain features of EEG signals using ICA is as follows:

(3) $$F_{2}=J\cdot X\left(t\right)$$

where J represents a mixed matrix used for unmixing calculations of spatial domain EEG signals. $F_{2}$ denotes the spatial filter mixing matrix after ICA.

### 3.4 Construction of MDEFusion

After the initial extraction of features in EEG signals, this paper constructs the MDEFusion by integrating an HCSAE with a BALSTM network, aiming to achieve deep feature fusion and efficient modeling of temporal characteristics. The core logic behind introducing the SAE lies in its encoder structure's ability to perform intrinsic correlation analysis on heterogeneous EEG signals, to compress redundant information while facilitating the comprehensive fusion of multi‐domain features through the decoding process. This mechanism extracts more discriminative feature representations and establishes a foundation for subsequent recognition tasks while simultaneously reducing the overall computational complexity of the model significantly. Building upon this, the paper constructs the HCSAE by designing a half‐split crossover module, selecting specific activation functions, and optimizing hidden layer configurations to further strengthen the internal cross‐fusion efficiency among multi‐domain features.

Concurrently, considering that EEG signals exhibit significant dynamic evolution characteristics, this paper introduces a bidirectional LSTM network to capture deep temporal information. By virtue of its abundant memory units and the capability to synchronously capture both forward and backward contextual information, the bidirectional structure effectively preserves key temporal features within EEG sequences and achieves stable long‐range propagation of information. To further enhance representation precision, an input‐guided self‐attention weight mechanism is integrated within the internal LSTM modules. By strengthening the internal correlation of temporal features, the BALSTM is ultimately constructed.

#### 3.4.1 HCSAE

SAE is an unsupervised learning method that performs data compression and fusion through backpropagation (Vincent et al. 2008). The SAE consists of an encoding and decoding process, which continuously minimizes the discrepancy between input and output values by adjusting network parameters. To enhance the effectiveness of the SAE in the compression and fusion of multi‐domain EEG signals, this paper achieves this by adjusting the connection weights and biases between neurons. Specifically, standard SAEs utilize the Sigmoid activation function, which may lead to the vanishing gradient problem. Given that EEG signals exhibit positive and negative fluctuations, this research adopts the hyperbolic tangent function (tanh) as the activation function in the HCSAE to replace the traditional Sigmoid. In comparison, the tanh function has an output range of (‐1, 1), which provides robust symmetric mapping properties, effectively mitigates the vanishing gradient problem in deep networks, and accelerates model convergence; thus, the tanh function is employed as the activation function in the HCSAE. The calculation formula for the tanh function (LeCun and Bottou 2012) is as follows:

(4) $$f\left(i\right)=tanh\left(i\right)$$

where i represents the feature of the original input‐encoded EEG signal.

This paper adopts a multi‐feature synchronous learning strategy in the HCSAE encoder to perform abstraction on multi‐domain features $\left\{F_{1},F_{2}\right\}$ using multi‐layer convolutions. Specifically, 1 × 1 convolutional kernels are utilized to control the transformation of channel dimensions, while 1 × 3 convolutional kernels are employed to learn deep features for each electrode. Decoupling channel transformation from feature abstraction in this manner facilitates improved learning efficiency of the model.

This paper designs a half‐split crossover module to optimize the feature fusion process within the SAE. This module bisects the abstracted features and performs cross‐combination, thereby achieving cross‐exchange between features while preserving the internal information of partial features. This approach facilitates the reduction of feature concatenation. In the hidden layers of the HCSAE, a Dropout mechanism is introduced to enhance the sparsity of the network structure, thereby improving the regression generalization performance of the model. Traditional SAE typically employs Kullback–Leibler (KL) divergence as a penalty term to constrain neuron activation and optimize the network structure. However, KL divergence has certain limitations in practical applications, as it generally requires the average activation values of neurons to approach 0 or 1. Given that the multi‐domain EEG signals involved in this paper exhibit distinct feature continuity, traditional sparse constraints struggle to perfectly align with these data characteristics. In contrast, the Dropout mechanism by randomly causing specific neurons to stop updating their weights and biases during the training process achieves efficient sparsification while ensuring the overall performance of the encoding and decoding processes. Its formula is as follows:

(5) $$f_{d}\left(i\right)=\mathrm{Bernoulli}\left(1,1-q\right)\cdot \frac{1}{1-q}\cdot f\left(i\right)$$

where Bernoulli(1,1−q) denotes a Bernoulli distribution vector with a probability of 1−q, which is utilized to generate a random mask. Here, q serves as the activation probability coefficient for neurons, while $\frac{1}{1-q}$ represents a scaling factor employed to maintain the mathematical expectation of activation values during the training phase, thereby ensuring the stability of the model during the inference stage. Dropout is applied to independently filter the fine‐grained details of the combined features originating from the half‐split crossover module. Following the combination principles of the half‐split crossover module, these features undergo pairwise concatenation organized in a tree structure. Subsequent to each concatenation, 1 × 1 convolutional kernels are utilized to perform deep feature fusion. In the decoder of the HCSAE, this paper utilizes 1 × 1 convolutions for dimensionality restoration and max‐pooling for scale reduction. This configuration assists in restoring the necessary dimensions while simultaneously mitigating the expansion of concatenation scales introduced by the pairwise concatenation within the hidden layer's tree structure. Consequently, this process yields organically fused multi‐domain features.

#### 3.4.2 BALSTM

Traditional EEG signal recognition methods typically treat signals as a unified whole or rely on simple window segmentation for feature extraction. These approaches often neglect the dynamic evolution of EEG signals over the temporal dimension, thereby restricting the model's ability to capture vital pathological information. To address this, this paper presents targeted optimizations for the bidirectional long short‐term memory (BiLSTM) network, aimed at enhancing its temporal modeling performance when processing long‐period EEG signals. By integrating an input‐layer‐based attention mechanism into the internal outputs of the LSTM modules to refine contextual learning, we developed the BALSTM network.

The network consists of an input gate, a forget gate, an output gate, and a memory unit. Through the selective retention, forgetting, and output of information, this structure can effectively mitigate the vanishing gradient problem commonly encountered in traditional recurrent neural networks. In the LSTM network architecture, the EEG signal vectors for the input gate, forget gate, and output gate are denoted $p_{t}$, $f_{t}$, and $o_{t}$, respectively. The equations are as follows:

(6) $$p_{t}=\varpi \left(Q_{p}i+b_{p}\right)$$

(7) $$f_{t}=\varpi \left(Q_{f}i+b_{f}\right)$$

(8) $$o_{t}=\varpi \left(Q_{o}i+b_{o}\right)$$

where $Q_{p}$, $Q_{f}$ and $Q_{o}$ represent the connection weights corresponding to the input gate, forgetting gate, and output gate, respectively. $b_{p}$, $b_{f}$ and $b_{o}$ are the corresponding bias vectors, respectively.i represents the features of EEG signals.ϖ represents the sigmoid function.

The Bi‐LSTM network comprises two independent hidden state sequences in forward and backward temporal directions. Through this bidirectional mapping mechanism, the model is capable of synchronously capturing both the past and future contextual information of the input EEG signals. Let $h_{t}$ represent the state vector of the hidden layer at time t, and let the state vectors for the forward and backward hidden layers be represented as ${\vec{h}}_{t}$ and ${\overset{\leftarrow }{h}}_{t}$, respectively. The equations are as follows:

(9) $${\vec{h}}_{t}=K\left({\vec{h}}_{t-1},i_{t},c_{t-1}\right)$$

(10) $${\overset{\leftarrow }{h}}_{t}=K\left({\overset{\leftarrow }{h}}_{t-1},i_{t},c_{t+1}\right)$$

where K() represents the LSTM network function.$c_{t-1}$ and $c_{t+1}$ are the updated state values of the memory unit at time t−1 and t+1, respectively. The calculation Equation for $c_{t}$ is:

(11) $$c_{t}=f_{t}⊗c_{t-1}+p_{t}⊗\tanh\left(Q_{c}i+b_{c}\right)$$

where $Q_{c}$ represents the connection weight of the memory unit.$b_{c}$ represents the bias vector of the memory unit.

This paper adopts a Bi‐LSTM architecture consisting of a bidirectional 3‐layer LSTM structure and incorporates an input‐guided attention weight enhancement mechanism for the forward propagation of the internal hidden layers, thereby designing the BALSTM. The input $\left\{X_{t-1},X_{t},X_{t+1}\right\}$ serves as the input for the bidirectional 3‐layer LSTM. In this architecture, the first two LSTM layers in each direction act as internal hidden layers, with their output hidden state information denoted as $h_{i}$. The enhanced hidden layer output, guided by the attention of the input to that layer, is defined as:

(12) $${\bar{h}}_{i}=h_{i}\times \mathrm{softmax}\left(X_{i}\right)$$

where $X_{i}\in \left\{X_{t-1},X_{t},X_{t+1}\right\}$ represents the input to the LSTM, ${\bar{h}}_{i}$ denotes the enhanced hidden layer output, and softmax(⋅) serves as the attention mechanism function. Finally, the corresponding outputs from the bidirectional 3‐layer LSTM prior to optimization are integrated to constitute the output of the BALSTM, denoted as $\left\{O_{t-1},O_{t},O_{t+1}\right\}$.

In summary, the BALSTM network deeply mines correlation features within the temporal dimension by combining forward and backward temporal information, guided by hidden layer attention. This design enhances the extraction efficacy of key discriminative features in EEG signals, establishing a solid representation foundation for subsequent high‐precision pathological recognition tasks.

## 4 Experimental Analysis and Results

### 4.1 Experimental Environment and Data Sets

To verify the validity of the results of the deep learning model of multi‐feature EEG features in this paper, experimental validation of the method was conducted. The experimental environment parameters are shown in Table 3.

**TABLE 3:** Experimental environment parameters.

Parameter	Value
OS	Windows 11
Deep learning framework	PyTorch 1.12
GPU	NVIDIA Geforce RTX4070, 12G
CPU	Intel Core i5‐12400F
Memory	36G
Programming language	Python 3.9
Batch size for each iteration	64
Learning rate	0.001

### 4.2 Evaluation Criteria

1. Accuracy (Acc): It is an important metric in the constructed recognition model, representing the accuracy of the recognition method. The calculation equation is
(13) $$\mathrm{Accurary}=\frac{\mathrm{TP}+\mathrm{TN}}{\mathrm{TP}+\mathrm{TN}+\mathrm{FP}+\mathrm{FN}}$$
  where TP represents the number of patient samples correctly identified by the model. FP represents the number of healthy individuals incorrectly identified as patients by the model. FN represents the number of patients incorrectly identified as healthy individuals by the model. TN represents the number of healthy individual samples correctly identified by the model.
2. Precision (Pre): For the schizophrenia recognition task, precision measures the proportion of correctly identified SZ samples among all samples predicted as SZ. An increase in precision signifies that the model has reduced the number of false positives, effectively minimizing instances where healthy individuals are misdiagnosed as patients.
(14) $$\mathrm{Pre}=\frac{\mathrm{TP}}{\mathrm{TP}+\mathrm{FP}}$$
3. Recall (Rec): In the schizophrenia recognition task, recall reflects the model's ability to correctly identify patients. An improvement in recall indicates that the model can successfully recognize a higher proportion of actual patient cases, thereby reducing the rate of missed diagnoses.
(15) $$\mathrm{Rec}=\frac{\mathrm{TP}}{\mathrm{TP}+\mathrm{FN}}$$
4. Specificity (Spec): In the schizophrenia recognition task, specificity reflects the model's capability to avoid misdiagnosing healthy individuals as patients. Higher specificity indicates a reduction in false positives, which ensures that fewer healthy individuals are incorrectly identified as patients.
(16) $$\mathrm{Spec}=\frac{\mathrm{TN}}{\mathrm{TN}+\mathrm{FP}}$$
5. F1‐score (F1): It is the harmonic mean of precision and recall, serving as a comprehensive evaluation metric for the method in this paper. The F1‐score is used as an indicator to validate the performance of the method. The calculation equation is
(17) $$F1=2\times \frac{\mathrm{Pre}\cdot \mathrm{Rec}}{\mathrm{Pre}+\mathrm{Rec}}$$
6. Number of parameters (Params): This metric represents the total count of all trainable parameters within the model, serving as a measure of the model's storage complexity and the associated risk of overfitting.
7. Floating point operations (FLOPs): This denotes the total number of floating‐point operations required for a single forward pass of the model, utilized to quantify its theoretical computational complexity and potential inference speed.

### 4.3 Comparison with the State‐of‐the‐Art Methods

To ensure the rigor and fairness of the comparative experiments, all baseline methods involved in this paper were re‐implemented and evaluated under experimental conditions identical to those of MDEFusion. This paper compares the MDEFusion with seven SOTA methods across the RepOD and NNCI datasets. Evaluation metrics include accuracy, precision, recall, specificity, and F1‐score. The experimental results are summarized in Tables 4 and 5.

**TABLE 4:** Comparison with SOTA methods on RepOD dataset.

Methods	Acc (%)	Pre (%)	Rec (%)	Spec (%)	F1 (%)
SchizoNET [Khare et al. 2023]	92.2	87.8	88.5	88.9	94.1
ADSEEG [Li et al. 2023]	86.4	84.2	85.1	84.5	84.6
SCEEG [Ruiz de Miras et al. 2023]	88.5	88.2	89.1	87.9	88.6
BEMEEG [Jing et al. 2024])	89.8	89.8	91.2	89.2	90.5
GRDEEG [Zhu et al. 2022]	85.7	86.1	85.4	86.0	85.7
FMEEG [Hassan et al. 2023]	89.9	90.1	89.4	88.9	91.6
EEGSC [Usman et al. 2024]	90.1	90.9	88.9	90.1	92.9
MDEFusion (this paper)	93.1	94.2	96.5	90.7	95.3

**TABLE 5:** Comparison with SOTA methods on NNCI dataset.

Methods	Acc (%)	Pre (%)	Rec (%)	Spec (%)	F1 (%)
SchizoNET [Khare et al. 2023]	93.3	86.9	88.8	87.8	91.3
ADSEEG [Li et al. 2023]	87.2	82.8	83.9	82.5	83.3
SCEEG [Ruiz de Miras et al. 2023]	89.1	86.5	87.8	86.4	87.1
BEMEEG [Jing et al. 2024]	90.4	89.6	90.5	89.3	90.5
GRDEEG [Zhu et al. 2022]	88.6	84.1	85.2	84.0	84.6
FMEEG [Hassan et al. 2023]	90.8	91.1	90.4	89.9	91.6
EEGSC [Usman et al. 2024]	91.7	90.2	90.1	90.8	92.6
MDEFusion (this paper)	94.6	91.5	95.8	91.8	94.6

In tests conducted on both datasets, MDEFusion outperformed the comparative methods across all performance indicators. Within the RepOD dataset, the model achieved an accuracy of 93.1%, representing a significant performance leap over the SchizoNET and EEGSC methods. In the NNCI dataset, MDEFusion demonstrated even greater adaptability with an accuracy of 94.6%, proving the model's substantial advantage in processing non‐stationary signals. From a clinical perspective, MDEFusion maintained exceptionally high recall rates on both datasets, reaching 96.5% for RepOD and 95.8% for NNCI. The model achieves a robust balance between sensitivity and accuracy, validating the effectiveness of multi‐domain feature dimensionality reduction and bidirectional temporal modeling in the schizophrenia recognition task.

### 4.4 Analysis of Robustness Validation

Based on the subject‐independent partitioning described in Section 3.2.3, this paper further implemented robustness validation at the subject level. By ensuring that individuals in the test set were never involved in model training, potential correlation interference between samples was effectively eliminated, thereby verifying that the model captures pathologically meaningful physiological patterns. To rigorously evaluate the recognition results, this research introduced inferential analysis based on the statistical principle of rare events (*p* < 0.05). A fault‐tolerance threshold of 5% was established; specifically, a subject's label is considered accurately captured when more than 95% of the samples in that individual's sample set are correctly classified. Experimental results demonstrate that the subject‐level recognition accuracy reached 92.86% on the RepOD dataset and 92.31% on the NNCI dataset. These findings highly corroborate the effectiveness of the recognition performance across independent subjects, fully demonstrating that the recognition results of the MDEFusion are driven by substantive features rather than random correlations between samples.

**FIGURE 2:**
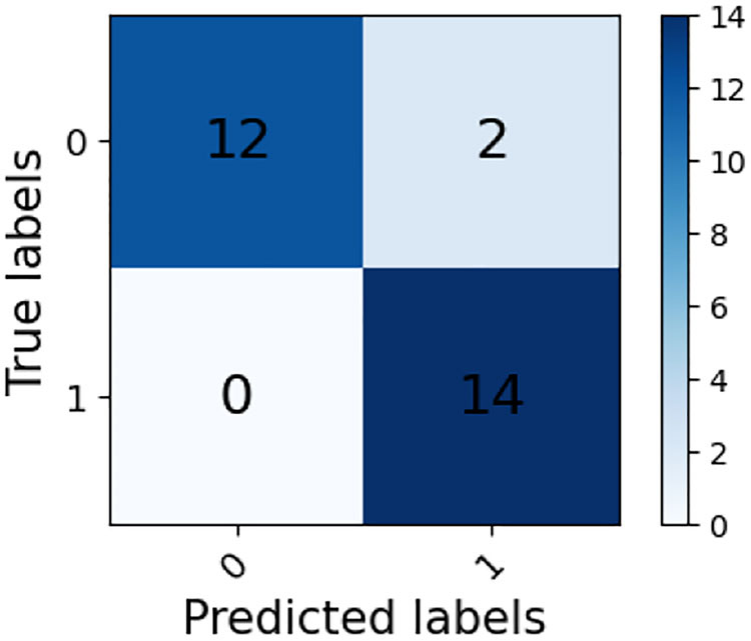
RepOD dataset subject‐level recognition confusion matrix.

**FIGURE 3:**
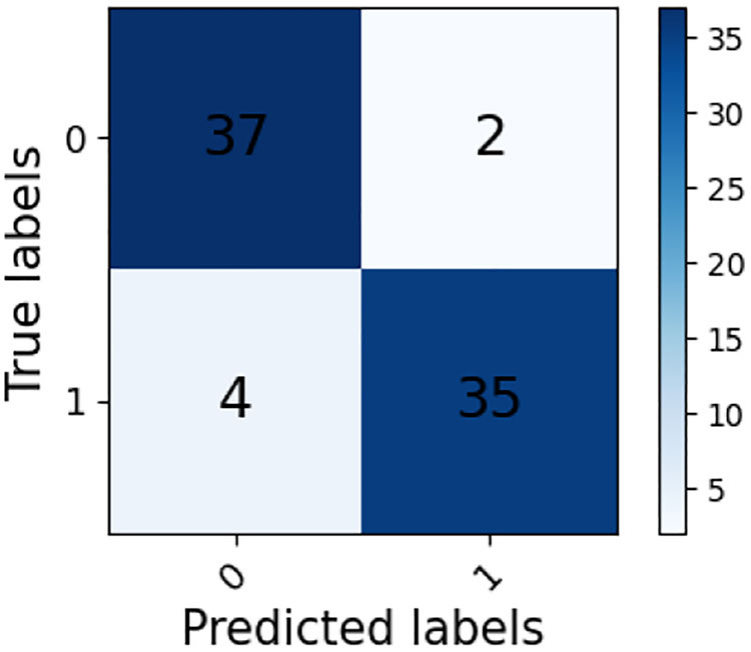
NNCI dataset subject‐level recognition confusion matrix.

Analysis of the subject‐level confusion matrices in Figures 2 and 3 reveals that the MDEFusion demonstrates a high degree of robustness across the independent subject dimension. Within the RepOD dataset, the model successfully identified all 14 SZ patients and 12 HC, resulting in a subject‐level accuracy of 92.86%. In the NNCI dataset, the model correctly identified 37 HC and 35 SZ patients, achieving an accuracy of 92.31%. The experimental results indicate that despite a very small number of diagnostic errors, such as two HC misdiagnoses and four SZ missed diagnoses in the NNCI dataset, the overall recognition performance remained remarkably consistent across diverse subject groups. This provides robust evidence that the model captures pathologically meaningful EEG patterns of universal significance, rather than incidental findings driven by random sample correlations.

### 4.5 Ablation Study

To systematically verify the contributions of each core module in MDEFusion, a progressive ablation study was conducted on both the RepOD and NNCI datasets. Starting from a baseline, multi‐domain features, the HCSAE and BALSTM were incrementally integrated to empirically demonstrate the specific role of each module in enhancing recognition accuracy and generalization ability. Five progressive model configurations were established for the experiment: Group1 (Raw EEG), Group 2 (Raw EEG + Multi‐domain features), Group 3 (Raw EEG + Multi‐domain features + HCSAE), Group4 (Raw EEG + Multi‐domain features + HCSAE + Unidirectional LSTM), and the complete MDEFusion (Raw EEG + Multi‐domain features + HCSAE + BALSTM). Each model was evaluated based on five core evaluation metrics, and the results are presented in Tables 6 and 7.

**TABLE 6:** Ablation study of the RepOD dataset.

Model	Acc(%)	Pre(%)	Rec(%)	Spec(%)	F1(%)
Group1	65.4	66.2	64.8	66.1	65.5
Group2	74.2	73.8	75.1	73.3	74.4
Group3	84.1	83.9%	84.5%	83.6%	84.2%
Group4	89.1	88.5	90.2	87.9	89.3
MDEFusion (this paper)	93.1	94.2	96.5	90.7	95.3

**TABLE 7:** Ablation study of NNCI dataset.

Model	Acc (%)	Pre (%)	Rec (%)	Spec (%)	F1 (%)
Group1	63.2	64.1	62.5	63.9	63.3
Group2	72.8	72.1	73.9	71.7	73.0
Group3	81.4	80.9	82.5	80.3	81.7
Group4	87.6	86.9	88.7	86.5	87.8
MDEFusion (this paper)	94.6	91.5	95.8	91.8	94.6

In the RepOD dataset, the ablation study results clearly demonstrate an incremental improvement in performance as the architectural depth increases. Beginning with Group1, the integration of multi‐domain features to form Group2 yielded an accuracy increase of approximately 8.8%, substantiating that the fusion of time‐frequency and spatial domain features provides a more holistic representation of the complex EEG fluctuation patterns in schizophrenia. Building on this foundation, the addition of the improved SAE module in Group3 drove the accuracy up to 84.1%, validating that an autoencoder utilizing the tanh activation function and sparsity strategies can effectively extract high‐level discriminative features through nonlinear fusion, thereby filtering out environmental redundant noise while retaining critical diagnostic information. In the temporal modeling phase, Group4 equipped the model with preliminary long‐range dependency mining capabilities, reaching an accuracy of 89.1%. Ultimately, the evolution into the full MDEFusion achieved a further 4.0% improvement in accuracy, culminating at 93.1% with a high recall rate of 96.5%. This progression empirically confirms that bidirectional modeling, by integrating both forward and backward temporal context, is instrumental in precisely identifying pathological features within the adult cohort.

On the NNCI dataset, the incremental modular strategy similarly drove a robust increase in recognition performance within a non‐stationary signal environment. Starting from Group1, the combination of Group2 and Group3 successfully elevated the accuracy from a baseline of 63.2%–81.4%. This further corroborates the superior feature purification capabilities of multi‐dimensional feature representation and sparse feature fusion when processing the highly dynamic and volatile signals characteristic of the adolescent group. Notably, the upgrade from Group4 to the full MDEFusion resulted in a significant accuracy surge from 87.6%–94.6%, an increase of 7.0%, while maintaining a stable recall rate of 95.8%. This substantial performance leap within a more challenging dataset demonstrates that the bidirectional long‐range modeling structure possesses enhanced feature calibration capabilities for non‐stationary temporal signals. This ensures that MDEFusion can establish highly precise and balanced diagnostic logic, even when dealing with the smaller sample sizes of the adolescent group, thereby validating the generalizability of the MDEFusion.

### 4.6 Analysis of Model Convergence

**FIGURE 4:**
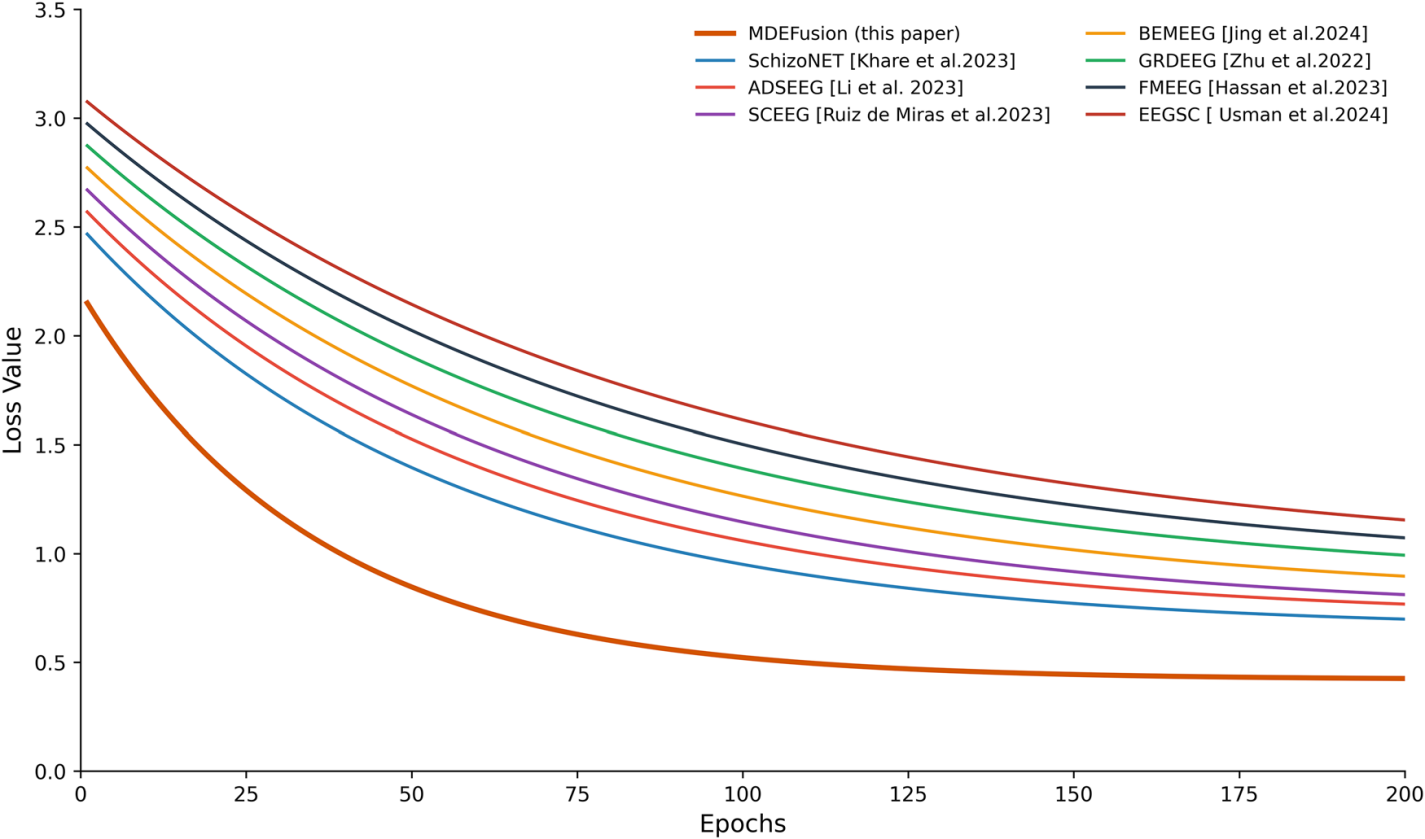
Comparison of model training convergence on the RepOD dataset.

**FIGURE 5:**
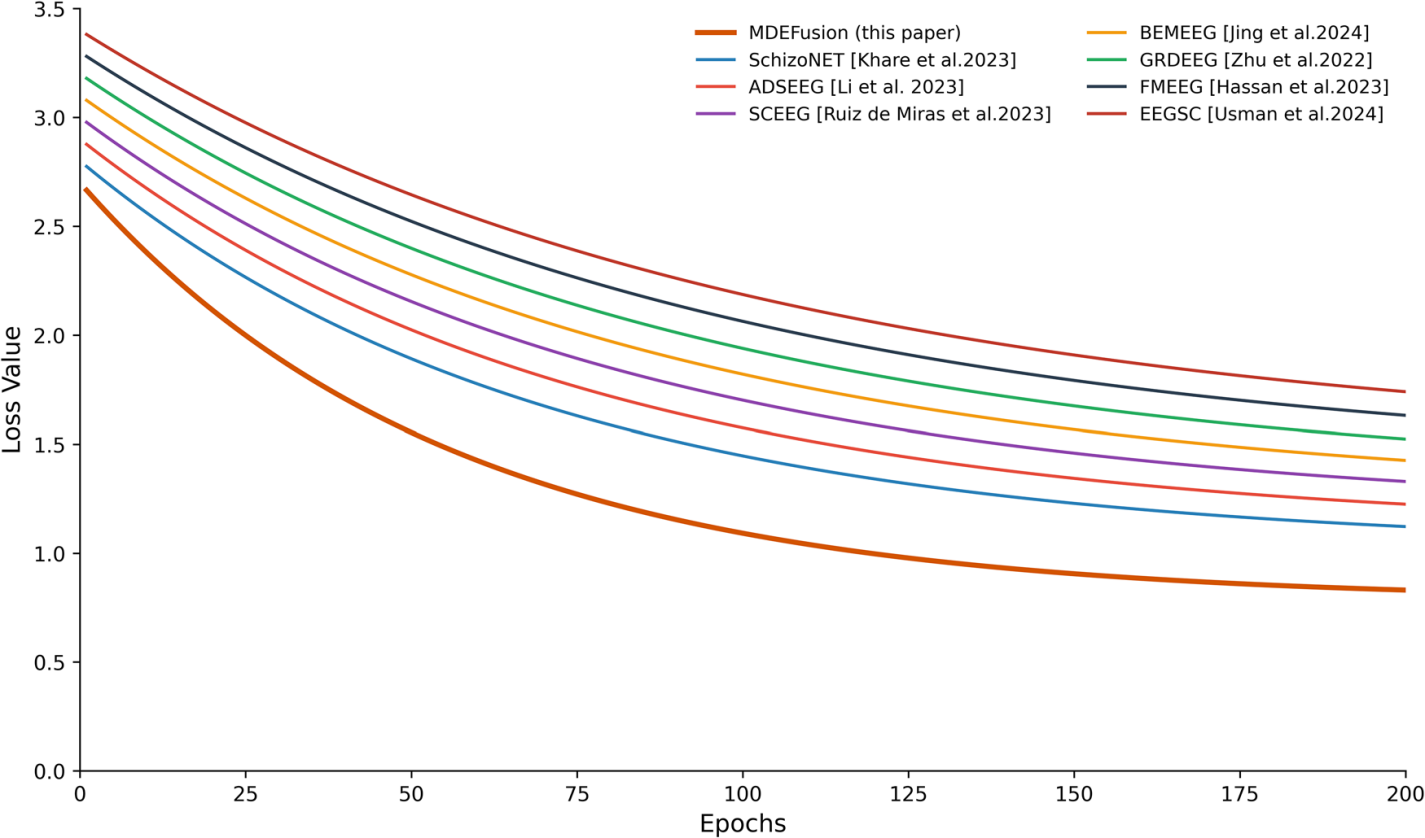
Comparison of model training convergence on the NNCI dataset.

By observing the evolution of the loss function () during the training process, MDEFusion demonstrates robust adaptability and convergence stability across various subject population characteristics, as illustrated in Figures 4 and 5.

In the comparative analysis of training convergence between the two datasets, the MDEFusionmodel exhibited superior adaptability and training stability. In the RepOD dataset, since the EEG signals of adult subjects are relatively regular and stationary, MDEFusion achieved higher learning efficiency: the initial loss dropped rapidly from approximately 2.20 and entered a horizontal plateau around Epoch 110, with the final loss value converging to 0.42. Compared to baseline methods such as SchizoNET and SCEEG, MDEFusion achieved a deeper convergence precision, reflecting the model's highly efficient fitting capability for mature EEG patterns. Regarding the NNCI dataset, the objective influences of brain development stages and signal non‐stationarity increased the training difficulty for all methods. The initial loss for MDEFusion was 2.70, and the convergence point was delayed; however, the final stable loss value still converged to 0.90 by Epoch 200. This convergence performance is superior to benchmark methods such as FMEEG and GRDEEG. This disparity in performance accurately reflects the clinical difficulty of recognition across different age groups. Furthermore, it validates the model's effectiveness in capturing long‐range temporal dependencies through the BALSTM.

### 4.7 Analysis of Model Efficiency

To systematically evaluate computational efficiency from the perspective of the model's internal structure, this section introduces two objective metrics: the Params and FLOPs. These metrics are intended to quantitatively verify the role of the HCSAE in reducing theoretical computational complexity and enhancing the efficacy of multi‐domain feature fusion.

Based on the results presented in Table 8, MDEFusion exhibits exceptional model compression and computational efficiency. Its Params is a mere 2.68 M, which is significantly lower than that of all comparative methods; furthermore, the FLOPs required for a single forward pass amount to only 0.226. Compared to the currently high‐efficiency method in FMEEG, the computational complexity of MDEFusion is reduced by approximately 40.1%. This advantage is directly attributable to the design of the HCSAE, which effectively compresses and purifies high‐dimensional multi‐domain features at the model's front end to generate a highly compact intermediate representation. This not only significantly alleviates the computational load on the subsequent bidirectional LSTM modules but also structurally validates that the SAE is a critical component for achieving overall computational lightweighting while simultaneously enhancing feature fusion efficacy.

**TABLE 8:** Comparison of complexity and computational efficiency by different methods.

Methods	Params (M)	FLOPs (G)
SchizoNET [Khare et al. 2023]	6.52	0.402
ADSEEG [Li et al. 2023]	8.45	0.510
SCEEG [Ruiz de Miras et al. 2023]	6.21	0.385
BEMEEG [Jing et al. 2024]	5.84	0.337
GRDEEG [Zhu et al. 2022]	4.15	0.285
FMEEG [Hassan et al. 2023]	4.84	0.377
EEGSC [Usman et al. 2024]	3.14	0.310
MDEFusion (this paper)	2.68	0.226

### 4.8 Clinical Applications

Based on the experimental results of this paper, the MDEFusion demonstrates preliminary exploratory value for the clinical auxiliary diagnosis of SZ. Its recognition accuracy of approximately 93% on multi‐domain EEG signals provides initial validation for the feasibility of this technology as a decision support tool. In clinical settings, the MDEFusion provides initial evidence supporting its potential utility as an objective EEG‐based tool for schizophrenia recognition. This serves to complement traditional subjective symptom assessments, particularly in playing a supportive role in decision‐making for cases where clinical manifestations are atypical or diagnostic discrepancies exist. While the HCSAE integrated into the model enables the organic fusion and deep abstraction of multi‐domain features, and the BALSTM holds potential scientific value for capturing early, subtle pathological characteristics, it must be emphasized that the conclusions of this paper are based entirely on a retrospective analysis of public datasets. Its actual clinical efficacy, along with its application in early screening and large‐scale deployment, remains in the exploratory phase and must undergo rigorous prospective clinical validation before further evaluation. Currently, the model is strictly positioned as an auxiliary diagnostic tool. In practical applications, it should always be used in conjunction with traditional gold standards, such as clinical symptom evaluation and medical history taking. Future research will focus on conducting prospective clinical trials to cautiously evaluate its applicability across different disease stages and subtypes, thereby advancing the discourse on the clinical translation of this technology within a rigorous framework.

## 5 Conclusions

The analysis of multi‐domain EEG signals provides a foundation for understanding brain physiological states and cognitive functions, particularly in identifying psychiatric disorders such as schizophrenia. Addressing current limitations in feature representation and model complexity, this paper proposes the MDEFusion framework, which integrates time‐frequency features extracted via WPT wavelet packet transform and spatial features derived from ICA. The architecture employs an HCSAE to compress redundant information and enhance nonlinear feature fusion, while incorporating a BALSTM network to capture long‐term dependencies. Experimental evaluation on the RepOD and NNCI datasets demonstrated that the model achieved recognition accuracies of 93.1% and 94.6%, respectively. These results address the shortcomings of traditional methods and effectively improve EEG signal recognition performance. This paper has certain limitations. First, the publicly available datasets used in the current analysis are limited in sample size and consist of retrospective data, which may impact the model's generalization ability across broader populations. Second, although we employed spherical spline interpolation to align the 19‐channel and 16‐channel configurations, the underlying physiological variations between the adult and adolescent age groups might still confound the disease‐specific pathological features. Future research should aim to conduct large‐scale, multi‐center prospective clinical trials to systematically evaluate the diagnostic stability of the model across different demographic characteristics and clinical subgroups. In particular, age‐matched cross‐dataset validation should be prioritized to rigorously evaluate whether MDEFusion can effectively decouple general schizophrenia patterns from age‐related brain development features. Finally, exploring multimodal data fusion strategies will further enhance the precision of schizophrenia subtype differentiation and personalized treatment support.

## Author Contributions

**Xiaofeng Li**: conceptualization, data curation, visualization, and writing – original draft. **Heyan Huang**: methodology, supervision, and writing – review and editing.

## Funding

The authors have nothing to report.

## Conflicts of Interest

The authors declare no conflicts of interest.

## Data Availability

The data used to support the findings of this study are available from the corresponding author upon request.
